# A description of a pre-emptive typhoid Vi capsular polysaccharide vaccination campaign after the 2015 earthquake in Nepal and vaccine effectiveness evaluation

**DOI:** 10.1186/s41182-024-00580-w

**Published:** 2024-01-29

**Authors:** Bhim Gopal Dhoubhadel, Ikumi Sawada, Dhruba Shrestha, Yoshifumi Fukuya, Ganendra Bhakta Raya, Eric Ipyn Nébié, Yumiko Hayashi, Rasila Pasakhala, Motoi Suzuki, Konosuke Morimoto, Christopher M. Parry, Koya Ariyoshi

**Affiliations:** 1 School of Tropical Medicine and Global Health, Nagasaki University, Nagasaki, Japan; 2 Department of Respiratory Infections, Institute of Tropical Medicine, Nagasaki University, Nagasaki, Japan; 3 Department of Clinical Medicine, Institute of Tropical Medicine, Nagasaki University, Nagasaki, Japan; 4 Department of Anesthesiology and Critical Care Medicine, Jichi Medical University Saitama Medical Center, Saitama, Japan; 5 Siddhi Memorial Hospital, Bhaktapur, Nepal; 6 Center for Infectious Disease, National Institute of Infectious Diseases, Tokyo, Japan; 7 Clinical Sciences and Education, Liverpool School of Tropical Medicine, Liverpool, UK

**Keywords:** Polysaccharide vaccine, Pre-emptive vaccination, Typhoid, Earthquake, Nepal

## Abstract

**Background:**

A 7.8 R scale earthquake hit Nepal in April 2015 and caused about 9000 deaths along with damage to infrastructure, including the water and sewage system. Bhaktapur was one of the highly affected districts. A typhoid vaccination campaign (pre-emptive) was carried out among children who were living in the temporary shelters in this district. The assessment of vaccine effectiveness after a pre-emptive typhoid vaccine campaign following an earthquake has previously not been attempted in Nepal.

**Objective:**

To describe the pre-emptive typhoid Vi capsular polysaccharide vaccination campaign and an evaluation of the vaccine effectiveness.

**Methods:**

We conducted a pre-emptive typhoid Vi capsular polysaccharide vaccination campaign among children between 2 and 15 years of age dwelling in 23 temporary shelters in Bhaktapur district after the earthquake. Surveillance of clinical typhoid was carried out from 2014 to 2017 in Siddhi Memorial Hospital, the only hospital for children in the district. We calculated vaccine effectiveness using a case–control study design (clinical typhoid as cases and chest x-ray confirmed pneumonia as controls).

**Results:**

Three thousand nine hundred sixteen children of age 2–15 years residing in the 23 temporary shelters in Bhaktapur received the typhoid Vi capsular polysaccharide vaccine between July and December 2015. 2193 children of age 2–15 years were admitted to the hospital during the study period and 260 (11.9%) were diagnosed with clinical typhoid. The numbers of children admitted with clinical typhoid decreased over the study period (105 in 2014 and 47 in 2017; *P* = 0.001). Overall vaccine effectiveness was calculated at 52% (95% CI −46 to 85%), and it was 87% (95% CI −25 to 99) among children less than 5 years of age.

**Conclusions:**

We successfully conducted a pre-emptive vaccination campaign against typhoid after the 2015 Nepal earthquake. The pre-emptive vaccination campaign appeared to be more effective among children less than 5 years of age. Further studies are needed to assess the effectiveness of pre-emptive use of typhoid vaccines in the emergency situations. We highlight the challenges of calculating vaccine effectiveness of a typhoid vaccine in an emergency setting.

**Supplementary Information:**

The online version contains supplementary material available at 10.1186/s41182-024-00580-w.

## Background

In April 2015, a 7.8 Richter scale earthquake hit Nepal causing about 9000 deaths and 2 million people displaced [1]. Fourteen out of 75 districts in the country were severely affected, and Bhaktapur in the Kathmandu valley was one of those [2]. Typhoid fever, a waterborne infection caused by *Salmonella* serovar Typhi, is endemic in Bhaktapur district, and due to damage to the water and sewage system, there was a potential risk of a typhoid outbreak after the earthquake [3–7]. The government and international organisations took steps to provide relief, with temporary shelters erected for those families who had lost their homes, and interventions and education to improve access to clean water and adequate sanitation.

Implementation of a vaccination programme to prevent an outbreak of an endemic disease in response to a disaster, or pre-emptive vaccination, is a further approach to prevent post disaster outbreaks [8]. Typhoid vaccines have been used pre-emptively in various typhoid endemic countries to prevent an outbreak of typhoid fever after natural disasters and are recommended by WHO [8, 9]. There is a paucity of typhoid vaccine effectiveness data when used pre-emptively after a natural disaster.

At the time of the 2015 earthquake there was no vaccination programme at the national level for typhoid in Nepal [10]. With the support of Nepal Paediatric Society and Siddhi Memorial Hospital in Bhaktapur, we organised a pre-emptive typhoid vaccination campaign with a Vi capsular polysaccharide vaccine (Typbar®), a licensed vaccine in the Department of Drug Administration (Government of Nepal), among children living in the temporary shelters in the district. Here we describe the implementation of this typhoid vaccination intervention and an attempt to measure vaccine effectiveness.

## Methods

### Vaccination campaign

Children aged between 2 and 15 years who were living in any of the 23 temporary shelters in Bhaktapur district were eligible for typhoid vaccination. The vaccination campaign was conducted between June and December 2015. Following an explanation of the intervention, and upon obtaining informed and signed consent from the parents, the Vi capsular polysaccharide vaccine Typbar^®^ (Bharat Biothech, India) was administered. Children were observed for a minimum period of one hour after vaccination to observe for the occurrence of adverse reactions.

### Assessment of vaccine effectiveness (VE)

The VE study was carried out in Siddhi Memorial Hospital (SMH). It is the only hospital that provides specialised paediatric care to children in Bhaktapur district. The temporary shelters were in the vicinity of the hospital which therefore acted as a healthcare facility for these children if they became sick. SMH has a total of 50 beds, including neonatal and paediatric intensive care units. The inpatient department has approximately 1500 admissions every year.

### Study populations

All children less than 15 years who were admitted to the paediatric inpatient department of SMH from January 1, 2014 to December 31, 2017 were included in this study. Children aged between 2 and 15 years of age were considered for the vaccine effectiveness analysis.

### Data collection

Inpatient data were obtained from patient’s hospital records. A standard, written pro-forma was completed at the time of discharge from hospital, or death, for each child admitted. Variables recorded included age, sex, admission date, clinical symptoms and signs, laboratory results (full blood count, Widal test, blood culture result, C-reactive protein), and diagnosis at discharge. The features suggesting clinical typhoid fever included some (but not necessarily all) of the following: a febrile illness of gradual onset and ≥ 3 days duration; the presence of abdominal symptoms (abdominal pain, diarrhoea, or constipation); a documented fever of ≥ 38 °C; hepatomegaly and/or splenomegaly; withdrawn or apathetic behavior; gastrointestinal bleeding; a low or normal white cell count; elevation of liver enzymes (AST, ALT) 2–3 times above the normal range; no other obvious focus of infection and where relevant a negative malaria blood smear. The limitation of blood culture for typhoid diagnosis is well recognised, which is related to a low number of circulating bacteria as well as prior antimicrobial treatment [12]. Clinical typhoid fever is indistinguishable from paratyphoid fever. Besides, other diseases that cause undifferentiated fevers, such as scrub typhus and leptospirosis may have similar clinical picture. Rapid diagnostic kits were used to rule out some of the undifferentiated fevers, including scrub typhus, leptospirosis, malaria, and dengue when necessary.

A group of the senior paediatricians approved a final diagnostic classification for each admission episode based on the clinical and laboratory data, and the clinical response to treatment. Children were classified with clinical typhoid fever, x-ray confirmed pneumonia, acute respiratory infection, acute gastroenteritis, and a febrile seizure according to the definitions in the Additional file 1: Table S1. The pro-forma data was entered in the Epi Info version 3.2 (CDC). Prospective data were collected from May 2015 to December 2017, and data from January 2014 to April 2015 were collected retrospectively from the hospital record. Typhoid vaccination status was checked from the vaccine campaign intervention records. The two datasets were merged using the child's name as the matching criterion. Upon merging the datasets, the names were removed, and a unique ID was allocated to ensure the anonymity and non-disclosure of individual identities. This allowed identification of vaccinated children who were subsequently admitted to SMH with clinical typhoid fever or an alternative diagnosis.

### Study design

A case–control study design was used to calculate the vaccine effectiveness. A case was a child with clinical typhoid fever defined as a child who presented with fever (≥ 38 °C) for 3 days or more with a confirmed culture result or clinically suspected to have typhoid fever. Children with chest x-ray confirmed clinical pneumonia were taken as controls.

### Data analysis

Data analysis was performed in Stata 15 (StataCorp, USA). Proportions were compared by the Chi-squared test or Fisher’s exact test. Vaccine effectiveness was calculated as [1—odds ratio (OR)] × 100%. Multivariable logistic regression models were used to calculate the adjusted vaccine effectiveness by adjusting potential confounders (age, sex, year of admission, CRP, and anaemia).

### Ethics

Informed written consent was taken from the parents of the children for their children to receive the typhoid vaccination. The vaccination campaign was expeditiously conducted as an emergency response, and as such, formal ethical approval was not sought. The vaccine effectiveness study was approved by the Institutional Review Board of Institute of Tropical Medicine, Nagasaki University (approval number: 15091740) and Nepal Health Research Council, Kathmandu, Nepal (Registered number 103/2017). The hospital data was collected passively as a routine hospital care, and this study was conducted retrospectively; therefore, only verbal consent was taken from the parents of the children whose anonymised data were used in the study. Both the IRBs waived the necessity of taking written consent for the vaccine effectiveness analysis.

## Results

A total of 3916 children of age between two and 15 years in the 23 temporary shelters in Bhaktapur were vaccinated with the typhoid Vi polysaccharide vaccine during the campaign between June 2015 and December 2015. Among the vaccinated children, 505 (12.9%) were under-5 years of age. Only 4 children (0.1%) had a mild adverse effect after vaccination: dizziness (n = 1), anxiety (n = 1), and pain with swelling at the site of injection (n = 2). All children with adverse effects were observed and treated by the attending paediatricians; these children did not develop any complications and later sent home. There was no case of serious adverse event following the immunisation. Characteristics of the vaccinated children are shown in Table 1.

**Table 1:** Characteristics of children vaccinated with typhoid Vi polysaccharide vaccine in temporary shelters after earthquake in Bhaktapur, Nepal

Characteristics	No of children n = 3916 (%)
Age, years	
< 5	505 (12.9)
≥ 5	3411 (87.1)
Sex	
Female	1957 (50.0)
Male	1959 (50.0)
History of previous vaccination against typhoid	
Yes	234 (6.0)
No	3682 (94.0)
History of taking treatment for typhoid in past	
Yes	209 (5.3)
No	3707 (94.7)
History of allergies	
Yes	187 (4.8)
No	3729 (95.2)
Adverse event following immunization (AEFI)	
Yes	4 (0.1)
No	3912 (99.9)

**Fig. 1:**
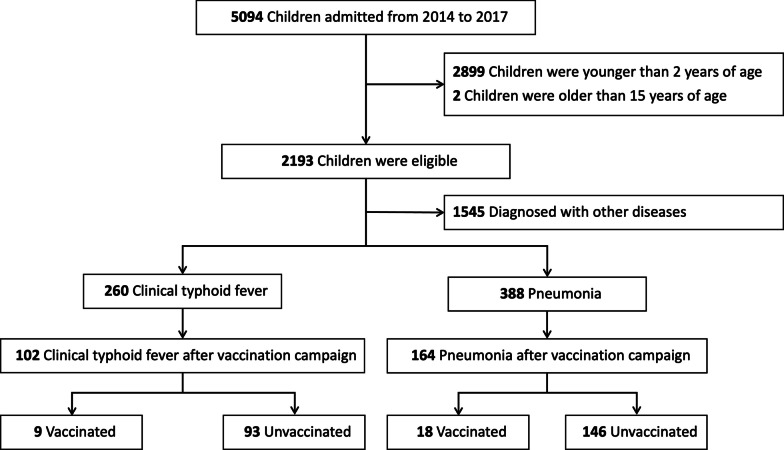
Flowchart showing the number of children admitted to the hospital during the study period

A total number of 5094 children were admitted to the inpatient ward of Siddhi Memorial Hospital during the study period from 2014 to 2017. The number of children aged between two and 15 years was 2193 with a median age of 48 months (IQR 31–84), and 59.9% were male (Table 2). Among the 2193 children, 260 were given a clinical diagnosis of typhoid fever, and 388 were diagnosed with chest x-ray confirmed pneumonia (Fig. 1). *Salmonella* spp., biochemically consistent with *Salmonella* Typhi, was isolated in 37 (3.0%) out of 1248 blood cultures. Out of 260 clinical typhoid fever cases, 151 tested positive on the Widal test.

**Table 2:** Demographic characteristics of children and distribution of common causes of hospital admission from 2014 to 2017

Characteristics/diagnosis	Total (n = 2193)	2014 (n = 605)	2015 (n = 489)	2016 (n = 557)	2017 (n = 542)	*P*-value
Male, n (%)	1314 (59.9)	374 (61.8)	300 (61.4)	322 (57.8)	318 (58.7)	0.439
Age in months, median (IQR)	48 (31, 84)	48 (30, 72)	48 (31, 84)	48 (30, 72)	49 (36, 84)	0.857
Typhoid fever, n (%)	260 (11.9)	105 (17.4)	53 (10.8)	55 (9.9)	47 (8.7)	0.001
X-ray confirmed pneumonia, n (%)	388 (17.7)	118 (19.5)	106 (21.7)	84 (15.1)	80 (14.8)	0.006
Acute respiratory infections, n (%)	423 (19.3)	99 (16.4)	64 (13.1)	128 (23.0)	123 (24.4)	0.001
Acute gastroenteritis, n (%)	140 (6.4)	31 (5.1)	31 (6.3)	42 (7.5)	36 (6.6)	0.405
Febrile seizure, n (%)	268 (12.2)	69 (11.4)	70 (14.3)	55 (9.9)	74 (13.7)	0.099
Others, n (%)	587 (26.7)	150 (24.8)	136 (27.8)	153 (27.5)	148 (27.3)	0.638

**Fig. 2:**
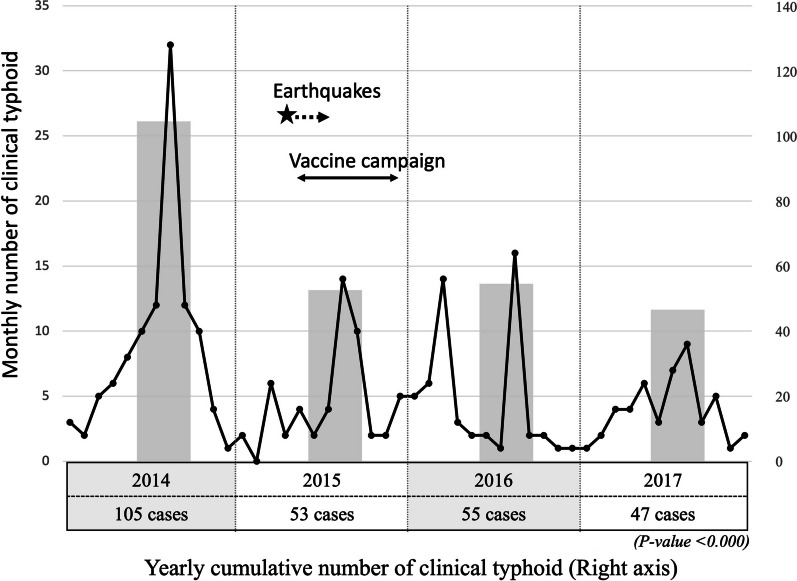
Line graph showing the number of children admitted to the hospital due to typhoid from 2014 to 2017. Grey bars show the cumulative number of clinical typhoid cases in high season of the year

The number of children diagnosed with clinical typhoid each month between 2014 and 2017 is shown in Fig. 2 and declined over the study period. Each year there was a peak in the number of children with clinical typhoid during the months of July to October. In 2016, there was a further peak in March.

The characteristics of children with clinical typhoid and children with chest x-ray confirmed pneumonia are compared in Table 3. The median age of children with clinical typhoid was 72 months, whereas it was 48 months for children with clinical pneumonia (*P* = 0.001). The numbers of children with clinical typhoid and clinical pneumonia declined over the study period. Clinical typhoid cases numbered 105 in 2014 and 47 cases in 2017 (Table 2, *P* = 0.001). Clinical pneumonia cases numbered 118 in 2014 and 80 in 2017 (Table 2, *P* = 0.006). The number of children admitted with acute gastroenteritis remained stable with 31 cases in 2014 and 36 cases in 2017 (Table 2, *P* = 0.405). Abdominal pain, vomiting, headache, and diarrhoeas were commonly observed in typhoid cases (Table 3). Cough and difficulty in breathing were more common in pneumonia (Table 3). Laboratory investigation data showed median WBC count was 7,000/mm^3^ in typhoid and 14,000/mm^3^ in pneumonia. Other laboratory findings are shown in Table 3.

**Table 3:** Comparison of characteristics of children with clinical typhoid and children with pneumonia in Siddhi Memorial Hospital in 2014–2017

Characteristics	Clinical typhoid fever (n = 260)	Pneumonia (n = 388)	*P*-value
Male, n (%)	159 (61.2)	237 (61.1))	0.985
Age in months, median (IQR)	72 (48, 108)	48 (30, 72)	0.001
Hospital stays, day, median (IQR)	6 (5, 7)	5 (4, 5)	0.001
Season of admission			
Mar–Jun	81 (31.2)	170 (43.8)	0.001
July–Oct	141 (54.2)	114 (29.4)	
Nov–Feb	38 (14.6)	104 (26.8)	
Symptoms			
Abdominal pain	43 (16.5)	39 (10.1)	0.015
Vomiting	41 (15.8)	39 (10.1)	0.030
Diarrhea	15 (5.8)	2 (0.5)	0.001
Cough	90 (34.6)	302 (77.8)	0.001
Difficulty of breathing	2 (0.8)	52 (13.4)	0.001
Headache	32 (12.3)	8 (2.1)	0.001
Laboratory findings			
WBC (× 10^3^/mm^3^), median (IQR)	7.0 (5.1, 9.5)	14.0 (9.4, 19.5)	0.001
Hb, g/dL, median (IQR)	12.1 (11.2, 13.0)	11.9 (11.0, 12.6)	0.021
Anemia*, n (%)	47 (21.2)	86 (23.9)	0.700
Platelets, median (IQR)	210 (165, 277)	277 (218, 359)	0.001
Elevated CRP (≥ 10 g/L)	149 (64.0)	276 (76.8)	0.001
Widal test, positive	151 (58.1)	6 (1.6)	0.001

Vaccine effectiveness (VE) was calculated by using the case–control study design. As the vaccination campaign was conducted in June to December 2015, data of 2016 and 2017 were used for VE calculation. The overall point estimate for adjusted VE was 52% (95% CI −46 to 84%). When this result was stratified by age, the adjusted vaccine effectiveness was higher among children younger than 5 years of age than in children older than 5 years of age (87% vs −10%) (Table 4).

**Table 4:** Effectiveness of ViPS vaccine against clinical typhoid in 2016–2017 after Nepal earthquake 2015

	Cases/controls (n)	Crude VE (95% CI)	Adjusted VE (95% CI)
Overall VE	102/164	22% (−82 to 66)	52% (−46 to 84)
Stratified analyses			
Sex			
Male	70/98	2% (−171 to 65)	41% (−158 to 86)
Female	32/66	52% (−142 to 90)	54% (−169 to 92)
Age groups			
< 5 years	37/94	26% (−186 to 81)	87% (−25 to 99)
≥ 5 years	65/70	21% (−141 to 74)	−10% (−372 to 75)

ViPS, Vi Polysaccharide vaccine; VE, vaccine effectiveness

## Discussion

We successfully implemented a pre-emptive typhoid vaccine campaign with the Vi capsular polysaccharide vaccine in children living in temporary shelters in one of the severely affected districts by the earthquake in Nepal in 2015. Nepal is endemic for typhoid and paratyphoid with an incidence of typhoid of 330 per 100,000 person-years and paratyphoid of 81 per 100,000 person-years in 2016–2019 [11]. Typhoid is common in Bhaktapur district and there was concern that, with the damage to sanitation infrastructure and interruptions in the availability of clean water, the displaced children might be at risk of a typhoid outbreak. During the campaign, we visited 23 temporary shelters in Bhaktapur and vaccinated 3916 children. Only four children had mild adverse event following the immunisation. The Vi capsular polysaccharide vaccine was chosen as it has been widely used, including in emergency situations, because it can be given as a single intramuscular dose, and has low cost and high heat stability [8, 12]. A limitation of the Ty21a oral attenuated vaccine is that it requires administration in three to four doses, and the new typhoid conjugate vaccine was not generally available or pre-approved by WHO at that time. Our experience showed that it was possible to conduct a typhoid vaccination campaign immediately after the earthquake with the support of governmental and non-governmental organisations. Nepal Paediatric Society (https://www.nepas.org.np/) advocated for the campaign and provided the technical support, and Siddhi Memorial Foundation (https://smf.org.np/) provided the logistic management.

The pre-emptive use of typhoid vaccination is a disease control strategy that should be considered in typhoid endemic regions to prevent an outbreak following a disaster [8, 13, 14]. We wanted to assess the effectiveness of this pre-emptive vaccine campaign in Bhaktapur district. The sole pediatric hospital in the area, SMH, implemented a simple prospective disease surveillance system in May 2015. Previous data from January 2014 to April 2015 were included in the dataset retrospectively. The surveillance data was used to conduct a retrospective epidemiological study of clinical typhoid and other causes of hospital admission for the period of 2014–2017.

We observed that the number of children admitted to the hospital with the diagnosis of clinical typhoid fever peaked between July and October each year. Patan Hospital, also in the Kathmandu valley where July is the month of highest rainfall, made similar observations of typhoid seasonality in a study from 2005 to 2009 [15]. We observed a significant decline in the number of clinical typhoid cases when comparing the period in 2014 before the earthquake and 2016–17 after the earthquake. There could be various reasons for this difference in addition to the typhoid vaccination intervention. Government and non-government organization initiated programmes to raise the awareness about safe water, sanitation, and hygiene in the earthquake affected regions, including Bhaktapur. This might have played a role in decreasing typhoid in the region although, of note, our data showed no decrease in children admitted with acute gastroenteritis over the same period. The fall in numbers may also have been part of an overall longer term decline in typhoid fever in the Kathmandu valley as was observed between 2005 and 2009 [15].

We calculated an overall vaccine effectiveness, using the case–control design, at 52% (95% CI −46 to 85%), and 87% (−25 to 99) among children less than 5 years of age. The confidence intervals were wide and included one, but the VE estimates were consistent with previous data. In a systematic review, one dose of Vi polysaccharide vaccine has an VE of 69% in the first year, 59–69% in the second year, and 55% in the third year of vaccination [16]. A large cluster-randomised trial (n = 37673) in India shows that VE of a single dose of Vi polysaccharide is 80% (95% CI 53–91) in children less than 5 years and 56% (95%CI 18–77) in older children [17].

The Vi capsular polysaccharide vaccine has been used pre-emptively following other disasters. In 2010, after a category 4 cyclone in Fiji, a typhoid vaccination campaign was carried out in the affected regions as a pre-emptive measure to prevent an outbreak. Following this intervention, a retrospective study showed that the incidence of typhoid decreased in post-campaign period in the vaccination areas (risk ratio 0.24, 95% CI 0.14–0.41) [9]. Typhoid vaccines have also been used pre-emptively after a cyclone in India in 2004 and after an earthquake in Pakistan in 2005 [8].

There are important limitations in our attempt to calculate vaccine effectiveness. The case definition was not specific for typhoid fever but was defined by clinical judgement of senior paediatricians. There was likely to have been a degree of consistency in the diagnosis because the same senior paediatric staff were responsible for clinical care during the whole study period. Only 14% of children with clinical typhoid fever had blood culture confirmed disease. The limitation of blood culture for typhoid diagnosis is well recognized and related to the low number of circulating bacteria in blood as well as prior antimicrobial treatment [12]. Similarly, the Widal test has limitations in diagnostic accuracy [12]. Clinical typhoid fever is indistinguishable from paratyphoid fever, for which the vaccine would not be effective. It is also possible that other diseases causing undifferentiated fever, such as scrub typhus and leptospirosis, may have caused a similar clinical picture. Mild and moderate cases of typhoid were treated in the outpatient department and were not included in this study. This contributed to the small sample size of cases. There might have been some data omissions due to similarities in the names of some children during the merging process of the two datasets.

In conclusion, we successfully conducted a pre-emptive Vi polysaccharide vaccination campaign to prevent an outbreak of typhoid after the 2015 earthquake in Nepal. Typhoid as a cause of hospital admission decreased significantly during the post-earthquake study period. Despite the data limitations, the point estimate of the calculated vaccine effectiveness was consistent with published results, and higher in children less than 5 years of age.

### Supplementary Information

**Additional file 1: Table S1.** Clinical case definitions.

## Data Availability

Upon a reasonable request, data related to this study are available from the corresponding author.
