# Supplementary material for: A description of a pre-emptive typhoid Vi capsular polysaccharide vaccination campaign after the 2015 earthquake in Nepal and vaccine effectiveness evaluation
Source: Trop Med Health. 2024 Jan 29;52:14. doi: 10.1186/s41182-024-00580-w (PMC10823638; doi:10.1186/s41182-024-00580-w)
Supplement: Supplementary file 1 — Additional file 1: Table S1. Clinical case definitions. [file 41182_2024_580_MOESM1_ESM.docx]

Additional Table 1. Clinical case definitions

| Clinical Diagnosis | Case definition |
| --- | --- |
| Clinical typhoid fever | Child who presented with fever (≥38°C) for ≥ 3 days; with at least two of the following symptoms - abdominal pain or headache or constipation/diarrhoea or weakness/fatigue or loss of appetite; and clinically suspected by the treating clinician to have typhoid fever. |
| Confirmed typhoid fever | Cases of clinical typhoid fever with a positive blood culture for *Salmonella* Typhi or *Salmonella* Paratyphi A or a positive Widal test (agglutination threshold of O antigen > 1:80). |
| Chest x-ray confirmed pneumonia | Children with lower respiratory tract symptoms and a chest x-ray with infiltrates |
| Acute respiratory infection | Children with lower respiratory tract symptoms and a chest x-ray with no infiltrates. |
| Acute gastroenteritis | Child with a sudden onset of three or more loose, watery, and unformed stools per day. |
| Febrile seizure | Child between 6 months and 5 years who had a single generalised convulsion associated with a high temperature (≥38°C) and full subsequent recovery. |
